# Applications of large language models in tumor boards: a systematic review

**DOI:** 10.3389/or.2026.1757059

**Published:** 2026-05-20

**Authors:** Benjamin Konzmann, Maurice Henkel, Christian Breit, Christian Wetterauer

**Affiliations:** 1 Institute of Urology, University Hospital Basel, Basel, Switzerland; 2 Digitalisation and ICT, University Hospital Basel, Basel, Switzerland; 3 Institute of Radiology, University Hospital Basel, Basel, Switzerland

**Keywords:** artificial intelligence in oncology, clinical decision support systems, large language models, multidisciplinary tumor board, precision medicine, systematic review

## Abstract

**Background:**

Multidisciplinary tumor boards (MDTs) are the gold standard for cancer care but currently face significant pressure from rising case volumes and the increasing complexity of precision medicine. Large Language Models (LLMs) offer potential as Clinical Decision Support Systems (CDSS) to augment these workflows. This systematic review evaluates the current applications, accuracy, and safety of LLMs in MDT decision-making.

**Methods:**

A systematic review was conducted following PRISMA 2020 guidelines. We searched PubMed/MEDLINE, Embase, IEEE Xplore, and arXiv for primary research published between January 2023 and November 2025. Inclusion criteria required studies to evaluate LLM performance in tumor board settings against human consensus or established clinical guidelines.

**Results:**

Thirty-one studies encompassing 3,845 unique patient cases were included. The analysis revealed a distinct “complexity gap” in model performance. In standardized, high-incidence domains such as breast and prostate cancer, advanced models (particularly the GPT-4 family) demonstrated high concordance (up to 94%) with human experts. However, performance degraded significantly in complex, rare, or multimodal scenarios—such as sarcoma or neuro-oncology—where models struggled with “gray zone” decision-making and lacked the ability to interpret non-textual data. While LLMs showed utility in administrative tasks and guideline retrieval, they remained prone to hallucinations and lacked the nuance required for holistic patient assessment.

**Conclusion:**

Current LLMs exhibit sufficient maturity to function as assistive tools for documentation and decision support in routine oncological cases but are not yet reliable enough for autonomous decision-making. Successful clinical implementation will require “human-in-the-loop” safeguards, the development of multimodal architectures, and rigorous prospective validation to ensure patient safety.

## 1 Introduction

Multidisciplinary tumor boards (MDTs) represent the gold standard for cancer care delivery, bringing together specialists from surgery, medical oncology, radiation oncology, pathology, and radiology to formulate evidence-based treatment recommendations (1). These collaborative forums face increasing pressure from rising cancer incidence, growing treatment complexity, and the rapid expansion of molecular data requiring interpretation (2). The integration of precision medicine has further complicated decision-making, with molecular tumor boards now tasked with interpreting genomic alterations, identifying targeted therapies, and matching patients to clinical trials (3).

To address this increasing complexity, researchers initially turned to classical Natural Language Processing (NLP) to optimize tumor board workflows through data structuring and aggregation. Prior to the widespread adoption of generative LLMs, tools like the “AI Pathway Companion” illustrated the potential of NLP-driven Clinical Decision Support Systems (CDSS) to enhance efficiency. Studies by (4, 5) demonstrated that these systems could significantly reduce case preparation times and improve clinician satisfaction in prostate cancer pathways by automatically extracting and presenting fragmented data from disparate clinical systems.

Building on these foundations, Large Language Models (LLMs) have emerged as the next-generation of tools to address these challenges, offering capabilities in natural language processing, pattern recognition, and knowledge synthesis that could augment human decision-making (6). Recent advances in transformer-based architectures, exemplified by GPT-4, Claude-3, and open-source alternatives like Llama, have demonstrated remarkable abilities to process medical literature, interpret clinical guidelines, and generate coherent clinical recommendations (7). However, the application of these general-purpose models to the highly specialized domain of oncology tumor boards raises critical questions about accuracy, safety, and clinical utility.

The potential benefits of LLM integration in tumor boards are substantial. These systems could standardize decision-making across institutions, reduce preparation time for complex cases, ensure comprehensive consideration of treatment options, and improve adherence to evolving guidelines (8). For resource-limited settings, locally deployed open-source models might democratize access to expert-level decision support (9). Additionally, LLMs could facilitate clinical trial matching, a labor-intensive process that currently results in only 3%–5% enrollment rates despite many eligible patients (10).

However, significant concerns mitigate this enthusiasm. The “black box” nature of LLM decision-making, potential for hallucinations, reliance on potentially biased training data, and medicolegal implications of AI-assisted recommendations all pose barriers to implementation (11). Furthermore, the dynamic nature of oncology, with rapidly evolving treatment paradigms and continuous publication of practice-changing trials, challenges models trained on historical data (12).

This systematic review addresses the current applications of LLMs in tumor board decision-making, compares AI-generated recommendations to human MDT decisions, assesses technical implementation requirements, and evaluates identified biases and safety concerns to determine the current technology readiness level for clinical deployment.

## 2 Methods

### 2.1 Protocol and registration

This systematic review was conducted following the Preferred Reporting Items for Systematic Reviews and Meta-Analyses (PRISMA) 2020 guidelines (Supplementary Table S2). The review protocol was developed *a priori*, specifying eligibility criteria, search strategy, and data extraction procedures. This review was not prospectively registered with PROSPERO or any other systematic review registry. We acknowledge this as a limitation; however, all eligibility criteria, search strategies, and data extraction procedures were specified prior to the literature search and followed throughout.

### 2.2 Eligibility criteria

Inclusion criteria encompassed primary research articles published in peer-reviewed journals or high-quality preprints between January 2023 and November 2025. Eligible studies evaluated LLM applications in oncology tumor boards or multidisciplinary team meetings and reported quantitative performance metrics comparing LLM recommendations to human decisions or guidelines. Only English-language publications were considered.

Studies were excluded if they were review articles, editorials, or opinion pieces without original data; utilized traditional machine learning or rule-based systems without LLM components; did not specifically address tumor board or MDT decision-making; or were conference abstracts without full-text availability.

### 2.3 Information sources and search strategy

The search was conducted across PubMed/MEDLINE, Embase, IEEE Xplore, and arXiv. The search strategy combined three concept groups using Boolean AND operators: (1) terms related to large language models (“large language model” OR “LLM” OR “ChatGPT” OR “GPT-4″ OR “GPT-3.5” OR “GPT-4o” OR “Claude” OR “Gemini” OR “generative artificial intelligence” OR “generative AI”), (2) terms related to tumor boards and multidisciplinary oncology (“tumor board” OR “tumour board” OR “multidisciplinary team” OR “multidisciplinary” OR “MDT” OR “cancer conference” OR “treatment decision” OR “treatment planning”), and (3) terms related to oncology (“cancer” OR “oncology” OR “neoplasm” OR “malignancy” OR “tumor” OR “tumour”). Database-specific search strategies with field tags and syntax adaptations are provided in Supplementary Table S1. The search was restricted to publications from January 2023 to November 2025. No language filter was applied at the search level; non-English publications were excluded during screening.

### 2.4 Data extraction and synthesis

Two reviewers (BK and CB) independently screened titles and abstracts, followed by full-text review of eligible articles. Disagreements at each stage were resolved through consensus discussion. Data extraction was performed by the first reviewer (BK) and captured study characteristics, cancer types, LLM models, clinical applications, and performance metrics. Due to substantial heterogeneity in study designs and outcome measures, a narrative synthesis was performed, grouping studies by primary application and cancer type. A quality assessment of included studies was conducted using a purpose-developed framework for LLM evaluation studies. Given the absence of established quality assessment tools for AI-assisted clinical decision-making research, we developed a novel ten-domain framework informed by principles of diagnostic accuracy assessment (QUADAS-2) but specifically tailored to the methodological challenges unique to LLM evaluation. The framework assesses: case selection method, case complexity spectrum, prompt reproducibility, repeat query testing, model parameter reporting, assessor blinding, independence of the reference standard, use of validated outcome metrics, error severity classification, and data realism. As this framework has not been externally validated, the quality assessment results should be interpreted as exploratory rather than definitive. Each domain was rated by the first reviewer (BK) and verified by the second reviewer (CB). A summary of the quality assessment is provided in Supplementary Table S4.

## 3 Results

### 3.1 Study selection

**FIGURE 1:**
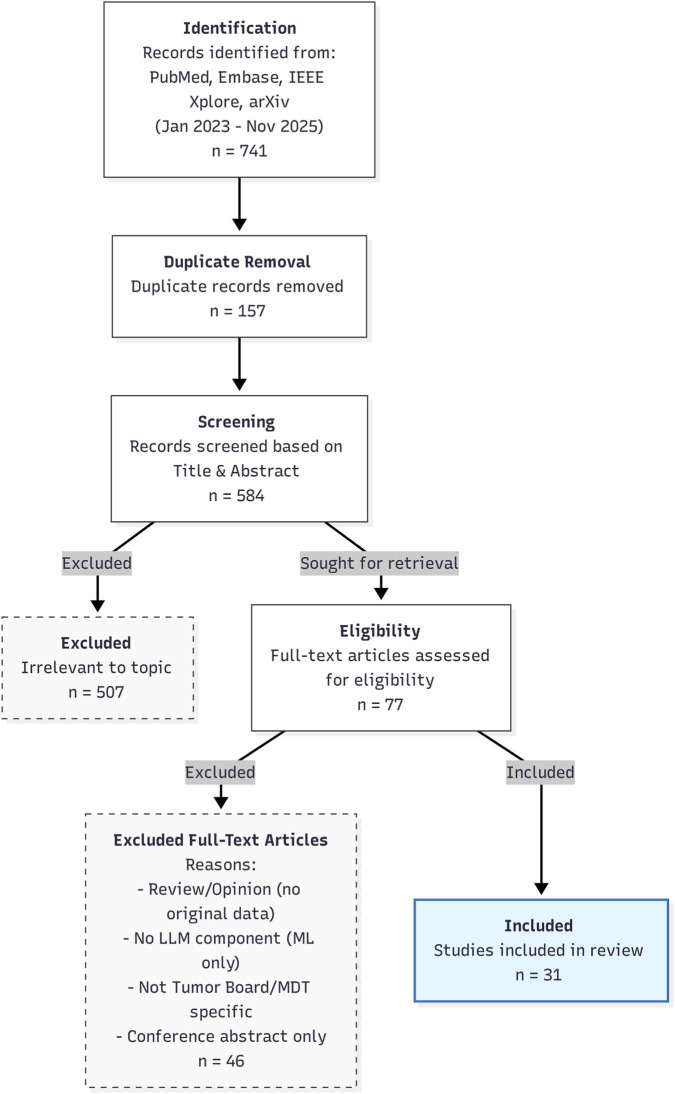
PRISMA Flow Diagram illustrating the literature search and study selection process. Flow diagram illustrating the systematic review process: 741 records identified, 157 duplicates removed, 584 records screened, 507 excluded as irrelevant, 77 full-text articles assessed for eligibility, 46 excluded for reasons (no original data, no LLM component, not tumor board/MDT specific, or conference abstract only), and 31 studies included in the review.

The initial database search yielded a total of 741 records. After removing 157 duplicates, 584 records remained for title and abstract screening. Two reviewers (BK and CB) independently screened these records, excluding 507 that did not meet the scope of the review (e.g., studies focused on traditional machine learning or rule-based systems rather than generative LLMs, studies addressing multidisciplinary teams outside of oncological decision-making, or studies not evaluating LLM performance in a tumor board or MDT context). The remaining 77 full-text articles were retrieved and assessed for eligibility. Of these, 46 were excluded for reasons including: lack of original data (review articles/opinion pieces), absence of an LLM component (traditional ML only), lack of specificity to tumor board/MDT decision-making, or being conference abstracts only. Ultimately, 31 studies met the inclusion criteria and were included in this review (Figure 1).

### 3.2 Study characteristics

This systematic review analyzed 31 studies published between 2023 and 2025, encompassing a total of 3,845 unique patient cases. The research landscape is global, with a notable concentration of studies in Germany (10 studies), followed by Turkey (4), Italy (4), and China (3) (Table 1). Study designs were predominantly retrospective comparative analyses, with the majority (29 of 31) evaluating LLM-generated recommendations against actual multidisciplinary tumor board decisions. Full study-level characteristics are provided in Supplementary Table S3. Of note, three included studies were preprints at the time of inclusion (Buyukceran et al., Kuerbanjiang et al., and (13)) and had not undergone formal peer review; these are clearly labeled in Supplementary Tables S3, S4, and their findings should be interpreted with this caveat in mind.

**TABLE 1:** Studies by country.

Country	No. of studies
Germany	10
Turkey	4
Italy	4
Mexico	2
China	3
Switzerland	2
France	2
Bosnia and Herzegovina	1
Israel	1
Singapore	1
Spain	1

Beyond binary concordance, studies employed a range of evaluation instruments including the AI Performance Instrument (AIPI), Likert-scale assessments for medical adequacy, Levels of Evidence classification, information density metrics, and consistency analyses across repeated queries. Two studies employed more advanced methods: Schmidl et al. (13) applied Kaplan–Meier and Cox proportional hazards models for survival analysis of LLM-selected versus tumor board–selected treatment cohorts, while Li et al. (14) conducted prospective validation through a ring trial across 21 sarcoma centers.

A total of 52 LLM instances (defined as unique model–study pairings, i.e., each distinct model version evaluated within a given study counts as one instance) were identified across the included studies. The OpenAI GPT family was the most extensively assessed (29 instances), spanning from ChatGPT-3.5 to GPT-4o and the reasoning model ChatGPT-o1. The Claude family accounted for 5 instances (Claude-2, Claude 3 Opus, Claude 3.5 Sonnet), the Llama family for 5 instances (Llama 2 through Llama 3.2-vision), and Google Gemini for 2 instances. Notably, specialized systems were also evaluated, including a guideline-augmented chatbot (TheSerenityBot, Claude-2 based), a trial-matching model (Trial-LLAMA), and the reasoning model DeepSeek-R1 in multi-agent configurations.

Breast cancer was the most frequently studied domain (11 studies), followed by head and neck cancer (6 studies), pan-cancer evaluations (4 studies), sarcoma (2), and molecular tumor board applications (2). The remaining studies addressed one domain each: genitourinary cancer, gynecologic oncology, lung cancer, neuro-oncology, prostate cancer, and general MDT settings (Table 2).

**TABLE 2:** Performance by specialty.

Specialty	Key findings and performance metrics
Breast cancer	Highly variable. Newer GPT-4/4o models showed high concordance (80%–94%) for standard treatments. Guideline-augmented models achieved 89% accuracy. Older models performed poorly (16%–50%). Complex decisions like genetic testing or specific anti-HER2 therapy were less accurate
Head and neck/ORL	Moderate to good for primary therapy (72%–96% concordance with newer models), but poor for detailed recommendations (e.g., second-line therapy 38%). Llama 3 was effective for curative/palliative distinction (92%)
Pan-cancer	Wide variability (low to moderate-high concordance). ChatGPT-4o showed strength in comprehensiveness but weakness in accuracy/feasibility in some studies
Sarcoma	Poor to moderate performance with safety concerns. Overall effectiveness ∼75%, but struggled with specific radiation recommendations and complex cases (20%–60% concordance)
CNS (neuro-oncology)	Good overall concordance (76%) but exhibited systematic overtreatment bias in intermediate “gray zone” scenarios, adopting a “when in doubt, treat” approach (18)
Genitourinary (GU) cancer	High concordance for prostate cancer (93%) but lower agreement for invasive procedures in RCC (12.9%)
Thoracic (lung) cancer	Good overall concordance (76%) with guideline incorporation, performing well for surgical recommendations (96% recall)
Gynecologic oncology	Multi-agent framework (DeepSeek-R1) achieved 98.26% accuracy, significantly outperforming single-agent configurations (∼88%)

### 3.3 Performance across clinical domains: The complexity gradient

An apparent pattern emerged across clinical domains, which we term the “complexity gradient”: LLM performance appeared to correlate inversely with the complexity of the clinical decision-making required. However, given the substantial methodological heterogeneity across studies—including differences in model versions, prompting strategies, and evaluation metrics—this pattern should be interpreted as an observational trend requiring prospective validation rather than an established causal relationship. In standardized, high-incidence domains with robust guideline-based algorithms, advanced models demonstrated high concordance with human MDTs. In breast cancer, GPT-4o achieved up to 93.9% concordance when using structured prompts and institutional protocols (15), and a prompt-driven AI assistant showed very good agreement for neoadjuvant therapy suitability (κ = 0.91) (16). In prostate cancer, Kaiser et al. (7) reported 93% alignment in pre-therapeutic decision-making using structured clinical data. In radiation oncology queries, ChatGPT-3.5 responses were on par with or superior to expert answers in 94% of cases (17). These findings suggest that for cancers with well-defined, algorithm-based guidelines, LLMs can effectively retrieve and apply standard-of-care protocols.

However, this reliability diminished in complex, rare, or nuanced clinical scenarios. In neuro-oncology, Tini et al. (18) found that while ChatGPT-4 aligned well with experts for clear-cut glioblastoma cases, it exhibited systematic overtreatment bias in intermediate “gray zone” scenarios. In sarcoma—a heterogeneous group of rare tumors—ChatGPT-4o showed variable performance across specialties with a mean Likert score of 3.76/5 (19), and even advanced models like Claude 3.5 Sonnet and OpenAI-o1 frequently failed to align with expert consensus in a multi-center ring trial (14). In laryngeal cancer, ChatGPT-4 provided recommendations consistent with MDT decisions in only 72% of patients (20), while in thoracic MDT boards overall concordance was 76% (κ = 0.59) (21). Across 102 patients with various cancer types, concordance between ChatGPT-4o and tumor board decisions remained low (22). This observed “complexity gap” suggests that current LLMs may excel at pattern matching within established guidelines but appear to struggle with the abductive reasoning required for the exceptions and edge cases that typically necessitate MDT discussion.

Beyond disease site, the specific nature of the clinical task influenced model reliability (Table 3). Treatment recommendation generation was effective in guideline-driven scenarios but degraded for complex or rare presentations. Guideline adherence checking achieved up to 100% accuracy when guidelines were explicitly integrated via retrieval-augmented generation. Clinical trial matching showed moderate improvement (+5% for molecular alteration extraction) (10), while staging classification depended heavily on the quality and structure of input data. Documentation and summarization tasks leveraged the natural language processing strengths of LLMs most directly.

**TABLE 3:** Performance by task.

Task	Performance summary	Why/Analysis
Treatment recommendation	**Works well in:** Standard, guideline-driven scenarios (e.g., breast, prostate) and high-level decisions (curative vs. palliative). **Struggles with:** Complex/nuanced patient factors, specific choices for advanced/rare cancers (sarcoma, second-line H&N), and invasive procedures	Lack of deep clinical reasoning and contextual understanding; inability to interpret multimodal data
Clinical trial eligibility	**Works moderately well for:** Refining molecular alteration extraction to improve trial matching (+5% improvement)	Good at specific text processing but overall matching remains complex
Staging classification	**Works well when:** Provided with structured inputs (e.g., TNM stages)	Dependence on data quality and structure
Guideline adherence checking	**Works well when:** Guidelines are explicitly integrated via RAG or structured prompts (up to 100% accuracy). **Struggles with:** Reliably citing sources without integration; can exhibit undertreatment bias	RAG/Prompt engineering significantly boosts retrieval accuracy compared to base model knowledge
Molecular data interpretation	**Works moderately well for:** Extracting information from text reports. **Struggles with:** Complex genomic interactions or rare mutations	High information density handling is good, but biological reasoning is limited
Documentation/Summarization	**Works well for:** Summarizing complex clinical situations and generating sensible decisions	Strong natural language processing capabilities utilized for administrative efficiency

Several studies identified specific sources of discordance beyond clinical complexity. Wang et al. (11) found that ChatGPT lagged significantly behind physicians in feasibility and appropriateness for ICU oncology patients, suggesting that models struggled with holistic patient assessment beyond guideline application. Umihanic et al. (23) observed that discordance frequently arose from the model’s inability to account for patient-specific contextual factors not captured in the structured prompt, such as frailty or local resource limitations. Additionally, the text-only nature of current LLMs created limitations in inherently multimodal disciplines: Sorin et al. (1) and Li et al. (14) noted that models relying on text reports rather than raw imaging data lacked the granular detail required for surgical planning and margin assessment.

### 3.4 Impact of model architecture and evolution

The review period (2023–2025) captured a rapid evolution in model capabilities. Earlier studies utilizing GPT-3.5 consistently reported lower concordance and higher hallucination rates compared to later investigations employing GPT-4 and GPT-4o. Griewing and Knitza (8) documented this trajectory, showing that the upgrade to GPT-4 significantly improved alignment with breast cancer tumor board decisions. Claude-3-Opus demonstrated superior comprehensiveness compared to GPT-4, achieving an F1 score of 0.95 for overall treatment recommendations in breast cancer (24). By contrast, Google’s Gemini showed significantly lower quality assessment scores compared to ChatGPT-4 in head and neck cancer (median QAMAI score 2 vs. 3, p = 0.004) and only slight agreement with MDT decisions in a pan-cancer evaluation (25).

Open-source and locally deployable models showed promise in specific niches. Llama-3 achieved 92% concordance for curative versus palliative treatment distinction in head and neck cancer, outperforming ChatGPT-4o in this specific task (9), suggesting that locally run models may offer viable decision support in resource-limited settings. The emergence of multi-agent frameworks represented a further architectural advance: DeepSeek-R1 deployed as part of a specialized agent team simulating different MDT specialists achieved 98.26% accuracy in gynecologic oncology treatment recommendations, significantly outperforming single-agent configurations (∼88%) (26).

### 3.5 Prompt engineering, retrieval augmentation, and reproducibility

How an LLM was queried proved as consequential as which model was used. Studies employing structured prompting, chain-of-thought reasoning, or retrieval-augmented generation (RAG) consistently outperformed zero-shot approaches. Buyukceran et al. (15) attributed their high concordance rates to a structured prompting strategy aligned with NCCN guidelines. A guideline-augmented tool (TheSerenityBot) based on Claude-2 with explicit NCCN context achieved 89% overall accuracy, outperforming both standalone Claude-2 (86%) and GPT-4 (78%) (27), illustrating the substantial gains from integrating authoritative clinical guidelines into the model workflow. Conversely, studies relying on unstructured case narratives often reported lower accuracy due to the model’s failure to identify missing critical information.

Reproducibility and response consistency emerged as significant concerns for clinical adoption. Liao et al. (28) found that only 32% of ChatGPT responses were fully consistent across repeated identical queries in a 362-patient breast cancer cohort, raising questions about the reliability of single-query evaluations. By contrast, Claude-3.5-Sonnet achieved 100% consistency across repeated queries in head and neck cancer cases (35), suggesting that response stability varies substantially across model architectures and may represent an important criterion for clinical deployment.

### 3.6 Quality assessment of included studies

The quality assessment revealed several systematic methodological limitations across the included studies (Supplementary Table S4). Case selection was generally adequate, with 21 of 31 studies using consecutive or retrospective cohort designs; however, five studies relied on simulated or fictional vignettes, limiting their external validity. Prompt reproducibility was a relative strength, with 23 studies publishing exact prompts or providing sufficient detail for replication. In contrast, only 9 of 31 studies tested the consistency of LLM responses across repeated identical queries—a critical gap given the stochastic nature of generative models. Model parameter reporting was notably deficient: only 3 studies fully specified inference parameters such as temperature, and 28 reported them only partially, with most omitting settings that directly affect output variability.

Assessor blinding was reported in only 8 studies, while 14 explicitly did not blind raters to whether recommendations originated from the LLM or the human MDT, introducing potential assessment bias. The independence of the reference standard was generally well-preserved (27 studies), as the retrospective design inherently ensured that MDT decisions preceded LLM evaluation. Most studies (24 of 31) employed validated outcome metrics such as Cohen’s κ, the AIPI, or Likert scales, though five relied solely on binary concordance percentages. Perhaps most consequentially, only 4 studies systematically distinguished between minor and clinically significant disagreements, meaning that a trivial dosing deviation and a fundamentally incorrect treatment modality could be coded identically as “discordant.” Twenty-three studies used real patient records, while five used simulated vignettes and three employed mixed or published reference data.

## 4 Discussion

This systematic review synthesizes findings from 31 studies evaluating the application of Large Language Models (LLMs) in multidisciplinary tumor board (MDT) decision-making. The cumulative evidence indicates that while LLMs have achieved a level of technical maturity sufficient to act as sophisticated clinical decision support systems (CDSS), they remain currently unsuited for autonomous deployment in complex oncological care. The analysis reveals a distinct dichotomy in performance: models demonstrate high concordance with human experts in guideline-driven, standard-of-care scenarios but exhibit significant performance degradation when navigating complex, rare, or multimodal clinical presentations. This discussion interprets these findings through the lens of model evolution, clinical nuance, and implementation challenges, outlining the trajectory for future integration into oncology practice.

### 4.1 Performance variability and the complexity gap

The most consistent observational pattern emerging from this review is an apparent inverse relationship between clinical case complexity and model performance. In standardized settings, particularly early-stage breast cancer and prostate cancer, LLMs demonstrated high concordance rates with human MDTs. For instance (15), reported near-perfect agreement (Mean 4.94/5) using GPT-4o with structured prompting for breast cancer, and (7) found 93% alignment in pre-therapeutic prostate cancer cases. Notably, however, even within breast cancer—the most extensively studied domain (11 of 31 studies)—reported concordance rates ranged from 16% to 94%, with the wide variation attributable to differences in model generation (GPT-3.5 vs. GPT-4o), prompting strategy (unstructured vs. guideline-aligned), and methodological rigor (34). These findings indicate that for high-incidence cancers with robust, algorithm-based guidelines (e.g., NCCN, ESMO), LLMs can effectively retrieve and apply standard protocols, though reported performance is highly sensitive to implementation choices.

However, this reliability fractures when models face “gray zone” cases, rare malignancies, or scenarios requiring nuanced risk-benefit analysis. In neuro-oncology (18), observed that while ChatGPT-4 aligned well with experts in clear-cut glioblastoma cases, it exhibited a systematic “overtreatment bias” in intermediate scenarios, adopting a “when in doubt, treat” approach that lacked the subtle clinical judgment regarding observation versus intervention in elderly or frail patients. Similarly, in the domain of sarcoma—a heterogeneous group of rare tumors—performance was notably poorer (14). demonstrated that even advanced models like Claude 3.5 Sonnet and OpenAI-o1 frequently failed to align with expert consensus in a ring trial of sarcoma centers, often hallucinating alternative recommendations that were clinically inaccurate. This observed “complexity gap” suggests a critical limitation: LLMs currently appear to excel at pattern matching within established text-based guidelines but struggle with the abductive reasoning required to manage the exceptions and edge cases that necessitate MDT discussion in the first place. Importantly, these concordance figures must be interpreted in the context of our quality assessment (§3.6): only 8 of 31 studies blinded assessors to the source of recommendations, and only 4 systematically distinguished clinically significant from minor disagreements, suggesting that reported concordance rates may both overestimate agreement and obscure the clinical relevance of errors. While the complexity gradient represents the most consistent observational pattern in our data, we caution that it is derived from cross-study comparison of heterogeneous methodologies rather than controlled experimental variation of case complexity. Prospective studies that systematically vary case complexity within a standardized evaluation framework are needed to confirm this relationship.

### 4.2 The evolution of model architectures

The review period (2023–2025) captures a rapid evolution in model capabilities. Early studies utilizing GPT-3.5 consistently reported lower concordance and higher hallucination rates compared to later investigations employing GPT-4, GPT-4o, and Claude 3.5 (8). documented this evolutionary trajectory, noting that the upgrade to GPT-4 significantly improved alignment with breast cancer tumor boards. Furthermore, the emergence of “reasoning” models, such as ChatGPT-o1, appears to influence how models handle staging and prognosis (13). noted that ChatGPT-o1 demonstrated a bias toward selecting early-stage cases with better survival outcomes, suggesting an advanced, albeit potentially biased, reasoning capability that differs from the pure probabilistic generation of earlier iterations. Perhaps most strikingly, the evolution extends beyond single-model improvements to novel architectural paradigms: multi-agent frameworks in which specialized AI agents simulate different MDT roles achieved 98.26% accuracy in gynecologic oncology, substantially outperforming single-agent configurations at approximately 88% (26). This finding suggests that the “complexity gap” identified in single-model evaluations may be partially addressable through system-level design rather than model scaling alone.

Despite these advancements, the “black box” nature of these models remains a persistent barrier to clinical trust. As noted by (30) in renal cell carcinoma and (31) in general oncology, the lack of transparency regarding how a model weighs conflicting evidence or specific patient factors precludes the validation necessary for medical liability. Unlike traditional rule-based CDSS where the logic path is transparent, LLMs generate outputs based on statistical probability, creating a “plausibility shield” where incorrect answers are presented with the same rhetorical confidence as correct ones. This was clearly illustrated by (12), who found that while ChatGPT 4.0 could reproduce guidelines, it frequently suggested lower-level evidence therapies (Level 3 or 4) as viable alternatives to standard care, potentially misleading clinicians in a real-world setting.

### 4.3 The human element: Context, comorbidities, and multimodality

A recurring theme across the extracted discussions is the inability of LLMs to fully capture the “human element” of MDT decision-making. Human tumor boards do not merely apply guidelines to tumor biology; they interpret these guidelines within the context of a specific patient’s physiological reserve, psychosocial status, and personal preferences. (11) highlighted that while ChatGPT provided comprehensive recommendations for ICU oncology patients, it lagged significantly behind physicians in feasibility and appropriateness, often suggesting interventions that were medically possible but practically inappropriate given the patient’s holistic status. Similarly (23), noted that discordance often arose because the AI failed to account for implicit knowledge—such as a patient’s frailty or local resource limitations—that was obvious to the human team but absent from the structured prompt. Across the 31 studies, this pattern is consistent: concordance was systematically higher in studies that pre-selected simplified or filtered case populations (e.g., untreated primary tumors, early-stage disease) than in those evaluating representative patient spectra. Our quality assessment (§3.6) confirms that fewer than half of included studies evaluated cases with representative clinical complexity—suggesting that the magnitude of the “human element” gap in routine clinical practice may be substantially greater than reported concordance rates imply.

Furthermore, the current generation of LLMs evaluated in these studies operates primarily on text, creating a “modality blind spot.” Oncology is inherently multimodal, relying on the synthesis of radiology, histopathology, and genomics (1, 14). emphasized that the inability of text-based models to directly interpret raw imaging or slide data forces them to rely on text reports, which may lack the granular detail required for surgical planning or margin assessment. While multimodal models (e.g., Llama 3.2-vision) are emerging (14), found that even when provided with selected images, these models could not replicate the surgical decision-making capacity of an expert board analyzing full radiological sequences.

### 4.4 Clinical utility: augmentation over automation

Given these limitations, the literature suggests the optimal role for current LLMs is augmentation rather than automation. Several studies identify high-value niches for immediate implementation (10). demonstrated that LLMs could improve the precision of clinical trial matching by 5% when processing molecular alterations, addressing a labor-intensive bottleneck in precision oncology. Additionally, the ability of LLMs to summarize complex patient histories and draft documentation is widely viewed as a mechanism to reduce administrative burden (7, 32).

In resource-limited settings, LLMs may serve a democratizing function (9). showed that locally run open-source models (like Llama 3) could achieve respectable concordance in head and neck cancer, potentially offering decision support in regions lacking specialized MDTs. However, this comes with the caveat of safety (33); warned, the use of these tools by non-experts without specialist oversight carries a risk of harm due to the models' tendency to hallucinate, overgeneralize, or misinterpret complex staging data.

### 4.5 Technical implementation and prompt engineering

The review highlights that how an LLM is queried is as critical as which model is used. Studies employing structured prompting, chain-of-thought reasoning, or Retrieval-Augmented Generation (RAG) consistently outperformed those using zero-shot prompts. However, our quality assessment (§3.6) reveals a critical transparency deficit: only 3 of 31 studies fully reported model parameters such as temperature and sampling settings, and only 23 provided sufficient prompt detail for full reproducibility—raising the possibility that reported performance differences may partly reflect unreported parameter variation rather than genuine methodological advantages (15). attributed their high concordance rates to a structured prompting strategy that aligned inputs with NCCN guidelines. Conversely, studies that relied on unstructured case narratives often reported lower accuracy due to the model’s failure to identify missing critical information (e.g., menopausal status in breast cancer). Furthermore, reproducibility emerged as a critical concern: as reported in Section 3.5, only 32% of ChatGPT responses were fully consistent across repeated queries (28), whereas Claude-3.5-Sonnet achieved 100% consistency (35). This variability in response stability, combined with the sensitivity to prompt design, underscores the necessity of standardized prompting protocols and rigorous reproducibility testing as prerequisites for clinical deployment.

### 4.6 Limitations of the review and included studies

The interpretation of these findings must be tempered by the limitations of the primary literature and the methodological quality of included studies (see §3.6). The majority of included studies (29 of 31) are retrospective comparative analyses. This design introduces “hindsight bias,” where the AI is judged against a decision already made, rather than evaluated in the dynamic, real-time environment of a tumor board where information unfolds progressively. Furthermore, there is a notable geographic bias, with a significant concentration of studies from Germany and Western Europe, potentially limiting the generalizability of findings to healthcare systems with different resource constraints or treatment guidelines. The quality assessment further identified that only 8 of 31 studies employed blinded assessment, only 9 tested reproducibility across repeated queries, and only 4 systematically classified error severity—meaning that the reported concordance rates may overestimate true clinical agreement and obscure clinically meaningful differences between minor and critical errors.

Methodological heterogeneity also complicates direct comparison. The metrics for success varied widely, from binary concordance (Agree/Disagree) to validated scales like the AI Performance Instrument (AIPI) or Likert scales for “medical adequacy.” As noted by Schmutz (2025), a simple concordance metric fails to capture the severity of an error; a minor deviation in chemotherapy dosing is clinically distinct from missing a surgical indication, yet both may be coded simply as “discordant” in some analyses. Additionally, many studies utilized “simulated” or “perfect” case vignettes which lack the messy, incomplete, and contradictory data characteristic of real-world oncology (9). Finally, this review was not prospectively registered, which may introduce concerns about potential protocol deviations, although the protocol was developed *a priori* and adhered to throughout. The quality assessment framework used in this review, while tailored to the specific characteristics of LLM evaluation studies, has not been externally validated; its domain weightings and scoring thresholds should therefore be interpreted with caution. No formal assessment of publication bias was conducted, as the predominantly narrative synthesis and heterogeneity in reported metrics precluded the use of standard funnel-plot or regression-based approaches. Similarly, although the breast cancer subgroup comprised 11 studies, a quantitative pooled analysis was not feasible due to substantial heterogeneity in concordance metrics, model versions, prompting strategies, and evaluation methods across these studies.

### 4.7 Implications for clinical practice and research

For clinical practice, the immediate implication is that LLMs should be viewed as “medical interns”—capable of summarizing data, checking guidelines, and drafting preliminary plans, but requiring strict supervision by senior clinicians. They are particularly useful for “sanity checking” routine decisions and ensuring comprehensive consideration of trial options (10). However, clinicians must remain vigilant against automation bias, where the coherent and authoritative tone of the LLM induces a false sense of security.

For research, the focus must shift from simple concordance studies to prospective, interventional trials. We need to understand not just if the AI agrees with the doctor, but if the AI improves the doctor’s decision or efficiency. Does the use of an LLM pre-meeting reduce the duration of the MDT? Does it increase the rate of clinical trial enrollment?

Based on the evidence synthesized in this review, we propose a tiered framework for LLM integration, stratified by clinical risk. For low-risk, administrative tasks—such as case summarization, documentation drafting, and guideline retrieval—the evidence supports near-term implementation with standard quality assurance oversight, as these tasks leverage core LLM strengths and carry minimal patient safety risk. For moderate-risk, guideline-driven decisions—such as standard-of-care recommendations in high-incidence cancers (e.g., early-stage breast or prostate cancer)—LLMs may serve as pre-meeting decision support tools, provided that outputs are systematically reviewed by qualified clinicians before being incorporated into patient care. For high-risk, complex decisions—including rare cancers, multimodal treatment planning, and cases involving significant patient-specific factors—current evidence does not support LLM use even in an advisory capacity without substantial human oversight, given the documented performance degradation in these scenarios.

The regulatory pathway for LLM-based clinical decision support remains undefined. Unlike traditional medical devices with predictable outputs, the stochastic nature of LLMs—evidenced by the low reproducibility rates documented in this review (32% full consistency for ChatGPT)—poses challenges for existing regulatory frameworks such as the FDA’s Software as a Medical Device (SaMD) pathway or the EU Medical Device Regulation (MDR). The lack of algorithmic transparency further complicates requirements for explainability. Future regulatory guidance will need to address questions of liability when AI-generated recommendations contribute to adverse outcomes, the classification of LLM-based tools within existing device categories, and the establishment of minimum performance and reproducibility standards for clinical deployment.

Cost-effectiveness data for LLM integration in tumor boards are conspicuously absent from the current literature. While proponents argue that LLMs could reduce MDT preparation time and increase throughput, no included study quantified time savings, resource utilization, or cost per patient. Future implementation studies should incorporate health economic evaluations to justify the infrastructure, licensing, and validation costs associated with clinical LLM deployment. Equally critical are data privacy concerns: the use of cloud-based commercial models (e.g., GPT-4, Claude) for processing patient data raises questions about HIPAA compliance, GDPR adherence, and institutional data governance. Locally deployed open-source models (e.g., Llama) may mitigate some privacy concerns but introduce challenges related to computational infrastructure and model maintenance. Any clinical implementation pathway must address data handling protocols, de-identification requirements, and institutional review processes.

### 4.8 Future directions

Future research should prioritize three interconnected domains. First, multimodal integration remains essential: developing and validating models capable of ingesting DICOM images and Whole Slide Images alongside clinical text would bridge the “modality gap” identified in sarcoma and surgical planning studies (14). Second, specialized “agentic” workflows warrant further exploration, moving beyond single-prompt interactions to multi-agent systems where different AI agents simulate different MDT specialists—an approach already showing promise in gynecologic oncology (26) and one that may better approximate the consensus-building process of a human tumor board. As agentic AI frameworks mature rapidly, their potential to improve decision-making in complex corner cases—such as synchronous double primary cancers or frail geriatric patients—where standard protocols often fail, represents a particularly promising avenue for future investigation. Third, real-world evidence and safety studies are urgently needed: prospective trials that integrate LLMs into the electronic health record workflow to evaluate performance on incomplete, contradictory clinical data, with specific attention to safety monitoring and the identification of potentially harmful hallucinations in rare disease subsets.

## 5 Conclusion

LLMs represent a promising but incompletely validated technology for oncology tumor boards, with emerging evidence suggesting potential to support care standardization, expand access to decision support, and reduce administrative burden. However, the current generation of models—evaluated exclusively in retrospective settings—exhibits a “capability ceiling” defined by case complexity and the lack of multimodal integration. While they have progressed from experimental curiosities to competent assistants for routine, guideline-driven cases, they are not yet reliable enough for autonomous decision-making in complex clinical scenarios. Based on the current evidence, the near-term role of LLMs in tumor boards lies not in replacement of expert deliberation, but in the careful design and prospective validation of human-in-the-loop systems that leverage the information-processing capabilities of LLMs while preserving the nuanced, context-dependent judgment of the multidisciplinary team.

## Data Availability

The original contributions presented in the study are included in the article/Supplementary Material, further inquiries can be directed to the corresponding author.
