# Supplementary material for: Applications of large language models in tumor boards: a systematic review
Source: Oncol Rev. 2026 May 20;20:1757059. doi: 10.3389/or.2026.1757059 (PMC13230141; doi:10.3389/or.2026.1757059)
Supplement: Supplementary file 1 [file Supplementaryfile1.docx]

# Supplementary Material

*Applications of Large Language Models in Tumor Boards: A Systematic Review*

## Supplementary Table S1: Database-Specific Search Strategies

Last search conducted: November 2025. Language restriction: none applied at search level (non-English excluded during screening).

| **Database** | **Search Strategy** | **Records** |
| --- | --- | --- |
| **PubMed/MEDLINE** | ("large language model" OR "LLM" OR "ChatGPT" OR "GPT-4" OR "GPT-3.5" OR "GPT-4o" OR "Claude" OR "Gemini" OR "generative artificial intelligence" OR "generative AI") AND ("tumor board" OR "tumour board" OR "multidisciplinary team" OR "multidisciplinary" OR "MDT" OR "cancer conference" OR "treatment decision" OR "treatment planning") AND ("cancer" OR "oncology" OR "neoplasm" OR "malignancy" OR "tumor" OR "tumour") Date filter: January 2023 – November 2025 | 297 |
| **Embase** | ('large language model':ti,ab OR 'LLM':ti,ab OR 'ChatGPT':ti,ab OR 'GPT-4':ti,ab OR 'GPT-3.5':ti,ab OR 'GPT-4o':ti,ab OR 'Claude':ti,ab OR 'Gemini':ti,ab OR 'generative artificial intelligence':ti,ab OR 'generative AI':ti,ab) AND ('tumor board':ti,ab OR 'tumour board':ti,ab OR 'multidisciplinary team':ti,ab OR 'multidisciplinary':ti,ab OR 'MDT':ti,ab OR 'cancer conference':ti,ab OR 'treatment decision':ti,ab OR 'clinical decision support':ti,ab) AND ('cancer':ti,ab OR 'oncology':ti,ab OR 'neoplasm':ti,ab OR 'tumor':ti,ab OR 'tumour':ti,ab) Date filter: January 2023 – November 2025 | 264 |
| **IEEE Xplore** | ("large language model" OR "ChatGPT" OR "GPT-4" OR "generative AI") AND ("tumor board" OR "tumour board" OR "multidisciplinary team" OR "clinical decision support") Year range: 2023–2025 | 82 |
| **arXiv** | Term 1 (All fields): ChatGPT OR "large language model" OR "GPT-4" OR LLM OR "generative AI" OR Claude OR Gemini AND Term 2 (All fields): oncology OR "tumor board" OR "treatment recommendation" OR "multidisciplinary team" Date range: 2023-01-01 to 2025-12-01; Include cross-listed papers | 98 |
| **Total records** |  | **741** |
| **Duplicates removed** |  | **157** |
| **Records screened** |  | **584** |

## Supplementary Table S2: PRISMA 2020 Checklist

| **Section/Topic** | **Item #** | **Checklist Item** | **Reported in** |
| --- | --- | --- | --- |
| Title | 1 | Identify the report as a systematic review | Title |
| Abstract | 2 | Structured summary with background, objectives, methods, results, limitations, conclusions | Abstract |
| Rationale | 3 | Describe the rationale for the review | §1 Introduction, para 1–4 |
| Objectives | 4 | Provide objectives with reference to PICO | §1 Introduction, para 5 |
| Eligibility criteria | 5 | Specify inclusion and exclusion criteria | §2.2 |
| Information sources | 6 | Describe all information sources and date of last search | §2.3; Table S1 |
| Search strategy | 7 | Present full search strategies for all databases | Table S1 |
| Selection process | 8 | Specify methods for selection and screening | §2.4 |
| Data collection process | 9 | Describe methods of data extraction | §2.4 |
| Data items | 10a | List and define all outcomes and variables | §2.4; Table S3 |
| Study risk of bias | 11 | Describe methods for assessing risk of bias | §2.4 (adapted QUADAS-2 framework); §3.6; Table S4 |
| Effect measures | 12 | Specify for each outcome the effect measure used | §3.2 (evaluation instruments) |
| Synthesis methods | 13a–13f | Describe synthesis methods | §2.4 (narrative synthesis) |
| Reporting bias assessment | 14 | Describe methods for assessing reporting bias | §4 Limitations |
| Certainty assessment | 15 | Describe methods for assessing certainty of evidence | Not applicable (narrative synthesis) |
| Study selection | 16a–16b | Results of search and selection with PRISMA flow diagram | §3.1; Figure 1 |
| Study characteristics | 17 | Characteristics of included studies | §3.2; Tables 1–3; Table S3 |
| Risk of bias in studies | 18 | Risk of bias assessment results | §3.6 Quality Assessment; Table S4 |
| Results of individual studies | 19 | Present results of individual studies | §3.3–3.5; Tables 2–3 |
| Results of syntheses | 20a–20d | Results of syntheses | §3.3–3.5 |
| Reporting biases | 21 | Reporting bias assessment results | §4 Limitations |
| Certainty of evidence | 22 | Certainty of evidence for each outcome | Not applicable |
| Discussion | 23a–23d | General interpretation, limitations, implications | §4 Discussion |
| Registration and protocol | 24a–24c | Registration information | §2.1 |
| Support | 25 | Sources of financial support | Funding Statement |
| Competing interests | 26 | Declarations of interest | Not reported |
| Availability of data | 27 | Template data collection forms, data extracted | Table S3 |

## Supplementary Table S3: Characteristics of Included Studies

| **Author** | **Year** | **Clinical Domain** | **Country** | **N** | **Models Evaluated** | **Design** | **Evaluation Method** | **Primary Task** | **Main Outcome** | **Prompt Strategy** |
| --- | --- | --- | --- | --- | --- | --- | --- | --- | --- | --- |
| Griewing S | 2023 | Breast Cancer | Germany | 20 | ChatGPT 3.5 | Retrospective | Direct MTB comparison | Treatment recommendation | 50-58.8% | Not specified |
| Lukac S | 2023 | Breast Cancer | Germany | 10 | ChatGPT (version not specified) | Retrospective | Direct MDT comparison | Treatment recommendation | Qualitative consensus | Not specified |
| Sorin V | 2023 | Breast Cancer | Israel | 10 | ChatGPT-3.5 | Retrospective | Direct MDT comparison | Treatment recommendation | 70% | Not specified |
| Yalamanchili A | 2023 | Pan-cancer (Radiation Oncology) | USA | 115 | ChatGPT 3.5 | Simulated | Domain-specific metrics | Documentation quality | 94% accuracy | Zero-shot |
| Griewing S | 2024 | Breast Cancer | Germany | 20 | ChatGPT 4, ChatGPT 3.5 (x2), Llama2, Bard | Retrospective | Direct MDT comparison | Treatment recommendation | 16-70.6% | Structured |
| Griewing S | 2024 | Breast Cancer | Germany | 20 | BC-SLM (tailored), ChatGPT 3.5, ChatGPT 4 | Simulated | Direct MTB comparison | Treatment recommendation | 83-90% | Structured |
| Lorenzi A | 2024 | Head & Neck Cancer | Italy | 5 | ChatGPT 4, Gemini Advanced | Simulated | AIPI / TDS scoring | Treatment recommendation | AIPI median 2-3 | Structured |
| Schmidl B | 2024 | Head & Neck Cancer | Germany | 100 | ChatGPT 4o, ChatGPT 4.0 | Retrospective | AIPI assessment | Treatment recommendation | AIPI-based (qualitative) | Not specified |
| Schmidl B | 2024 | Head & Neck Cancer | Germany | 50 | Claude 3 Opus, ChatGPT 4.0 | Retrospective | AIPI / Direct MDT | Treatment recommendation | Qualitative (AIPI) | Structured |
| Ah-thiane L | 2025 | Breast Cancer | France | 112 | Claude 3 Opus, GPT4-Turbo, LLaMa3-70B | Retrospective | Direct MDT comparison | Treatment recommendation | 77.7-86.6% | Structured |
| Ammo T | 2025 | Sarcoma | Germany | 5 | ChatGPT-4o | Simulated | Panel assessment (Likert) | Treatment recommendation | 3.76/5 mean Likert | Structured |
| Bertolo R | 2025 | Genitourinary Cancer | Italy | 103 | ChatGPT o1 (GPT-4.1) | Retrospective | Direct MDT comparison | Treatment recommendation | 62.1% (k=0.44) | Structured |
| Buhr CR | 2025 | Head & Neck Cancer | Germany | 25 | ChatGPT-4o, Llama 3 | Prospective | Direct MDT comparison | Treatment recommendation | 84-92% | Structured |
| Buyukceran E ^*^ | 2025 | Breast Cancer | Turkey | 33 | GPT-4o | Retrospective | Direct MDT comparison | Treatment recommendation | 93.9% | Structured |
| Cossu M | 2025 | Breast Cancer | Italy | 30 | ChatGPT (AI assistant) | Retrospective | Direct MDT (Cohen's k) | Surgery planning | k=0.29-0.91 | Structured |
| Dogan I | 2025 | Pan-cancer (General Oncology) | Turkey | 100 | ChatGPT-4.0 | Prospective | Direct MDT (Cohen's k) | Treatment recommendation | 76.4% | Structured |
| Goh SSN | 2025 | Breast Cancer | Singapore | 50 | TheSerenityBot (Claude-2 based), Claude-2, GPT-4 | Retrospective | Direct MTB consensus | Treatment recommendation | 78-89% | RAG |
| Gueguen L | 2025 | Molecular Tumor Board (Pan-cancer) | France | 157 | Klineo, ScreenAct, Trialing, DigitalECMT (LLM-enhanced) | Prospective | Trial matching evaluation | Trial matching | 45% (AP@3) | Not specified |
| Hernandez-Flores LA | 2025 | Pan-cancer (General Oncology) | Mexico | 98 | ChatGPT 4o, Gemini 1.5 Flash | Retrospective | Direct MDT (Cohen's k) | Treatment recommendation | k=0.024-0.525 | Not specified |
| Kaiser P | 2025 | Prostate Cancer | Switzerland | 171 | ChatGPT-4, Claude-3-Opus | Retrospective | Direct MDT comparison | Treatment recommendation | 93% | Structured |
| Karabuga B | 2025 | Pan-cancer (General Oncology) | Turkey | 102 | ChatGPT-4o | Retrospective | Direct MDT comparison | Treatment recommendation | k=0.211-0.376 | Structured |
| Kuerbanjiang W ^*^ | 2025 | Gynecologic Oncology | China | 182 | ChatGPT-4o, DeepSeek-R1, Llama-4 | Retrospective | Multi-agent framework | Treatment recommendation | 88-98.26% | Multi-agent |
| Li CP | 2025 | Sarcoma | Germany | 5 | Llama 3.2-vision:90b, Claude 3.5 Sonnet, DeepSeek-R1, OpenAI-o1 | Simulated | Direct MTB (ring trial) | Treatment recommendation | 20-60% | Structured |
| Liao N | 2025 | Breast Cancer | China | 362 | ChatGPT-4.0 | Prospective | Direct MDT comparison | Treatment recommendation | 46% | Not specified |
| Pamuk E | 2025 | Head & Neck Cancer | Turkey | 25 | ChatGPT-4 | Retrospective | Direct MDT comparison | Treatment recommendation | 72% | Not specified |
| Schmidl B ^*^ | 2025 | Head & Neck Cancer | Germany | 1757 | ChatGPT o1, ChatGPT 4o | Retrospective | Survival analysis + MDT | Survival analysis | HR-based (no sig. diff.) | Structured |
| Schmutz M | 2025 | Molecular Tumor Board (Pan-cancer) | Germany | 20 | ChatGPT 4.0 | Retrospective | LoE comparison | Molecular interpretation | Fleiss k=0.51 | Structured |
| Tini P | 2025 | Neuro-oncology / CNS | Italy | 101 | ChatGPT-4 | Prospective | Direct MDT comparison | Treatment recommendation | 76% | Structured |
| Umihanic S | 2025 | Breast Cancer | Bosnia and Herzegovina | 91 | ChatGPT-4.0 | Retrospective | Direct MDT comparison | Treatment recommendation | 82.4% | Structured |
| Wang X | 2025 | General MDT (Critical Care / ICU) | China | 64 | ChatGPT | Retrospective | Direct MDT comparison | Treatment recommendation | 82% | Structured |
| Zabaleta J | 2025 | Lung Cancer | Spain | 52 | GPT 3.5 turbo | Retrospective | Direct MDT comparison | Treatment recommendation | 76% (k=0.59) | Structured |

^*^ Preprint (not peer-reviewed at the time of inclusion). Buyukceran et al. and Schmidl et al. (2025) were posted on Research Square; Kuerbanjiang et al. was posted on medRxiv.

## Supplementary Table S4: Quality Assessment of Included Studies

Quality assessment was conducted using a framework adapted from QUADAS-2 for LLM evaluation studies. Ten domains were assessed for each study. Ratings were assigned by the first reviewer (BK) and verified by the second reviewer (CB).

| **Study** | **Case Selection** | **Case Complexity** | **Prompt Reprod.** | **Repeat Queries** | **Model Params** | **Blinding** | **Indepen- dence** | **Validated Metric** | **Error Severity** | **Data Realism** |
| --- | --- | --- | --- | --- | --- | --- | --- | --- | --- | --- |
| Ah-thiane L (2025) | Consecutive | Representative | Yes | No | Partial | Partial | Yes | Yes (F1, specificity, accuracy) | Partial | Real records |
| Ammo T (2025) | Simulated cases | Representative spectrum | Yes | No | Partial | No | N/A (simulated) | Yes (5-point Likert) | Partial | Simulated |
| Bertolo R (2025) | Consecutive | Representative | Yes | No | Yes (temp=0.30, top-p=1.0) | No | Yes | Yes (Cohen's κ, Fisher's exact) | Partial | Real records |
| Buhr CR (2025) | Simulated cases | Designed mix | Yes | No | Partial | No | Unclear (simulated) | Yes (6-point Likert) | Partial | Simulated |
| Buyukceran E (2025) ^*^ | Consecutive | Representative | Yes (supplementary) | Yes (2 sessions, identical results) | Partial | Yes | Yes | Yes (Likert, Cohen's κ, F1) | Partial | Real records |
| Cossu M (2025) | Consecutive | Mixed | Yes | No | Partial | No | Yes | Yes (Cohen's κ) | No | Real records |
| Dogan I (2025) | Consecutive | Mixed (pan-cancer) | Yes | No | Partial | Yes | Yes | Yes (Cohen's κ, Spearman ρ) | Partial | Real records |
| Goh SSN (2025) | Consecutive | Representative | Yes | No | Partial | No | Yes | Yes (GEE, F1, accuracy) | Partial | Real records |
| Griewing S (2023) | Fictional vignettes | Systematically varied | Yes | Yes (randomized sequence) | Partial | Yes (partial) | Yes | No (descriptive % only) | Partial | Simulated |
| Griewing S (2024) | Fictional vignettes | Systematically varied | Yes (supplementary) | No | Partial | No | Yes | No (binary concordance only) | No | Simulated |
| Griewing S (2024) | Fictional vignettes | Comprehensive spectrum | Yes (published prompting model) | No | Yes (detailed: Mixtral 8x7B, A6000 GPU) | Partial | Yes | Yes (Cohen's κ) | No | Simulated |
| Gueguen L (2025) | Consecutive | Representative | Partial | No | Partial | Unclear | Yes | Yes (precision, AP@k, nDCG@k) | Partial | Real records |
| Hernandez-Flores LA (2025) | Consecutive | Mixed | Partial | No | Partial | No | Yes | Yes (Cohen's κ) | No | Real records |
| Kaiser P (2025) | Consecutive | Representative | Yes | No | Partial (API, cutoff dates) | No | Yes | No (% agreement, Fisher's exact) | Partial (3 error categories) | Real records |
| Karabuga B (2025) | Convenience (complex cases) | Deliberately complex | Partial | No | Partial | No | Yes | Yes (weighted Cohen's κ, Likert) | Partial (Likert gradient) | Real records |
| Kuerbanjiang W (2025) ^*^ | Mixed (synthetic + case reports) | Mixed | Partial | No | Partial (temp=0.2 for generation) | Yes (double-blind case eval) | N/A (synthetic + case reports) | Yes (6-dim Likert, r=0.894) | Yes (harm-weighted scoring) | Mixed (synthetic + case reports) |
| Li CP (2025) | Ring trial cases | High complexity | Yes (supplementary) | Yes (21x per case) | Partial (temp=0 where possible) | N/A (algorithmic comparison) | Yes (prior ring trial consensus) | Partial (concordance metrics) | Partial | Real records (ring trial) |
| Liao N (2025) | Consecutive | Representative | Partial | Yes (3x by different users) | Partial | Unclear | Yes | Yes (custom 5-point scale, Cohen's κ) | Partial | Real records |
| Lorenzi A (2024) | Convenience (5 vignettes) | Simplified | Partial | No | Partial | Yes | Yes (vs NCCN guidelines) | Yes (TDS, AIPI) | Yes (TDS severity grades) | Simulated (based on real cases) |
| Lukac S (2023) | Consecutive | Simplified (early stage only) | Yes | Yes (2 sessions) | Partial | No | Yes | No (custom scoring 0-400) | No | Real records |
| Pamuk E (2025) | Consecutive | Filtered (untreated primary only) | Yes | No | Partial | Partial | Yes | No (custom 4-grade scale) | No | Real records |
| Schmidl B (2024) | Consecutive | Representative | Yes | No | Partial | Yes (double-blind) | Yes | Yes (custom 5-point scale, Cohen's κ) | Partial | Real records |
| Schmidl B (2024) | Consecutive | Mixed | Partial | No | Partial | No | Yes | Yes (AIPI, Cohen's κ) | No | Real records |
| Schmidl B (2025) ^*^ | Consecutive (retrospective cohort) | Representative | Yes | Yes (3x per case) | Partial | No | Yes | Partial (% agreement + Cox models) | No | Real records |
| Schmutz M (2025) | Consecutive | Representative (heterogeneous) | Yes (supplementary transcripts) | Yes (triplicates, Fleiss' κ) | Partial | No | Yes | Yes (LoE, Fleiss' κ, IDM) | Partial (severity weighting proposed) | Real records |
| Sorin V (2023) | Consecutive | Limited (mostly IDC) | Partial | No | Partial | Partial | Yes | Yes (Cohen's κ, Likert) | No | Real records |
| Tini P (2025) | Consecutive | Stratified by complexity | Yes | Yes (30-day interval, κ=0.96) | Yes | Yes | Yes | Yes (Cohen's κ, McNemar's test) | Yes (over/undertreatment bias) | Real records |
| Umihanic S (2025) | Consecutive | Representative | Yes | No | Partial | Partial | Yes | Yes (Likert, Cronbach's α, Fleiss' κ) | No | Real records |
| Wang X (2025) | Consecutive | Mixed (ICU) | Yes (supplementary) | No | Partial | Yes | Yes | Yes (5-point Likert) | No | Real records |
| Yalamanchili A (2023) | Expert-selected questions | Stratified by question type | Yes (exact wording) | No | Partial | No (by design) | N/A (published expert answers) | Yes (Likert, cosine similarity) | Yes (potential harm scale 0-4) | Published Q&A (not patient data) |
| Zabaleta J (2025) | Consecutive | Mixed (data quality issues) | Yes | Yes (4 iterations) | Partial | Unclear | Yes | Yes (Cohen's κ) | Partial | Real records |

^*^ Preprint (not peer-reviewed at the time of inclusion).
